# Mr. Bransby Cooper's Lectures on Anatomy, Vol. IV

**Published:** 1833-01-01

**Authors:** 


**Lectures on Anatomy. Interspersed with Practical Remarks. Vol. IV. By *B. B. Cooper*, F.R.S. Surgeon of Guy's Hospital, &c. &c. &c. Large Octavo, pp. 383, four Plates, Longman's, London, 1832.**

This is the concluding volume of Mr. Bransby Cooper's System of Anatomy. He has redeemed the pledges which he gave, and the work is now complete. We have on former occasions spoken in a very favourable manner of the three first volumes, and yet we have not bestowed on them more commendation than they merited. The volume before us does no dishonour to its predecessors, in fact it may be looked on as the best. We expressed our belief that Mr. Cooper's Anatomy would prove a popular one, and our anticipations have been fulfilled. Now that it is completed, we entertain no doubt that it will become a general favourite with students. We have given samples of the other volumes, and in justice we should do the same by this. They must be taken in a great degree at random, and therefore may not be the best that could be selected. In order to illustrate the descriptive portion we may just insert Mr. Cooper's short account of the spinal sinuses and vertebral vein, the former of which, strangely enough, are seldom noticed in manuals of anatomy.

"*Ramus venosus vertebralis*—is made up of ramuli corresponding to the distributing ramuli of the arterial ramus. It commences principally by small ramuli from muscles in the occipital and posterior cervical regions, and there forms itself into a large trunk, which takes its course with the vertebral artery upon the posterior circular ligament between the atlas and occiput; it passes forwards to gain the foramen at the root of the transverse process of the atlas, near to which it is united with the lateral sinus of the dura mater, by a venous ramulus, which passes through the posterior condyloid foramen. The ramus venosus vertebralis then descends through the canal formed by the transverse processes of all the cervical vertebræ, being accompanied by its corresponding artery. In this course it anastomoses internally, through the intervertebral foramina with the sinus venosus; and externally, it receives in each intervertebral space, venous ramuli from the muscles of the neck. This ramulus issues from the canal of the seventh cervical vertebra, and descends by the side of its accompanying arterial ramulus, between the rectus capitis anticus major on its inner side, and the scalenus anticus on the outer. In this space receiving venous ramuli returning the blood from the ramulus cervicalis profundus; then it passes, on the left side before the subclavian artery, and on the right side behind that vessel, to terminate in the subclavian vein." 137.

*Ramuli venosi spinales, vel sinus vertebrales*—take the course of the whole of the vertebral canal, from the sacrum to the occiput, behind the bodies of the vertebræ, anterior to the theca vertebralis, and on the sides of the common proper posterior ligament. They commence from the venous ramusculi in the adipose tissue of the sacral canal, and are augmented by ramusculi from the dura mater, enveloping the spinal marrow along the whole length of the spinal column. In this course they anastomose with the venous ramusculi of the lateral sacral, lumbar, and intercostal rami, on their outer sides; and on their inner sides, by short vessels with each other, on the bodies of the vertebræ, underneath the posterior vertebral ligament, where they receive ramusculi from the substance of the bodies of the vertebræ.

These ramuli venosi are called sinuses, because they have short communicating cross-tendinous bands in them, similar to the sinuses of the dura mater. They communicate with the rami jugulares interni, opposite to the anterior condyloid foramina.

The ramuli venosi spinales, appear to contract opposite to the fibro-cartilages, and to enlarge upon the bodies of the vertebræ, hence they have a knotted appearance, which is more particularly observable in the lumbar region." 145.

The following is a specimen of the physiology of the nervous system.

" To M. Serres, we are indebted for the light which he has thrown upon the cause, which immediately regulates the growth of the whole nervous system; and this he has found to depend upon the distribution of the arteries. Thus he has observed, that the spinal marrow is produced by the intercostal arteries, which are the first formed blood-vessels in the fœtus; the cerebellum, by the vertebral; and the cerebrum, by the carotid arteries. In these formations, the crura cerebri and tubercula quadrigemina, follow that of the spinal marrow; the cervical region of which is first formed, by the branch of the superior intercostal artery, which enters the vertebral canal. The vertebral artery arrives the last within the cranium, and the cerebellum is therefore the last organ apparent in the formation of the encephalic organ of all classes. Following the arterial order of nervous development, it is observed that the cerebellum is formed from behind forwards, and the cerebrum, on the contrary, from before backwards; according, in the first instance, with the vertebral artery, which enters at the posterior part, and is directed from behind forwards; and in the latter instance, with the internal carotid artery, which after passing the turns of the cavernous sinus, reaches the anterior part of the brain, and proceeds from before backwards, according to the progress of the formation of the cerebrum.

Hence also the corpus callosum, and anterior part of the fornix, are developed from before backwards, following the progress of the artery; while the posterior part proceeds from behind forwards, with the posterior cerebral artery.

In animals, the development of the cerebrum and cerebellum, results from the general relation in this union of the vertebral and carotid arteries; and the comparative size of the brain and spinal marrow, appear to depend upon the respective calibre of the arteries which supply these parts with blood. Thus when the spinal arteries are large, the more the spinal marrow is developed; and the more the vertebral and internal carotids increase in size, the greater is the development of the brain. And it is remarkable, that these structures bear an inverse ratio in their formation, which does not depend upon the preponderance of any portion of the nervous matter in the spinal marrow, but entirely upon that law of life which has regulated the distribution and course of the sanguineous system.

This, as a general principle, is applicable to the nerves in all parts of the organismus.

Thus the great sympathetic nerve diminishes in proportion to the decrease of the sanguineous system.

The size of the middle sacral artery, regulates the dimensions of the caudal prolongation in all animals.

The appearance of the extremities, and their nerves, also depend upon the calibre of their respective arteries: thus the axillary arteries in bimana, and the femoral in bipeds, are those which attain the fullest development. So in the head, the development of the face and the brain depend on the volume of the external carotid in the former, and of the internal carotid in the latter. Where the external carotid is large, the face and nerves of sense are extensive.

The ophthalmic artery, however, is always in proportion to the size of the external carotid; from which circumstance, the volume of the eye is still regulated according to the extent of the organs of smell and taste.

Tracing the formation of the nerves themselves, their first appearance is in the form of globules of medullary matter, deposited in the cellular and mucous tissues; these increase, and unite in centres of communication, which form the ganglia: from the ganglia, cords communicating with each other, are next seen taking the course of the larger blood-vessels. This organized state, is first apparent in the semilunar ganglion; and after the ganglia, then the centres of communication of the voluntary nerves appear; and lastly, the nerves destined for the higher powers of voluntary motion and sensation." 168.

M. Serres' reasonings do not appear to us to be conclusive. It may be true, and no doubt it is so, that the volume of the nervous mass bears a relation to the size of the artery, but it does not therefore follow that the efficient cause of the magnitude or otherwise of the brain or medulla exists in the arterial development. When a tumour springs up the blood-vessels enlarge, but the enlargement of the blood-vessels was not the cause of the tumour; on the contrary, it is more reasonable to suppose that its growth necessitating an increased supply of blood, was the cause of the augmentation of the vessels. The final cause of all such processes is evident, the efficient cause has ever baffled human scrutiny. The sanguineous system being the system of supply, there must necessarily be a consent between the natural growth or unnatural development of parts and the media of supply. If there were not, the economy would not be what it is, but the different systems composing the body are so dovetailed with each other, that it is scarcely philosophical to give precedence to any in the operations of growth. At the same time that we feel indisposed to agree with M. Serres altogether in his theory, the facts which he mentions are highly deserving of attention.

The only other topic to which we can advert is Mr. Cooper's notice of M. Foville's investigations into the anatomy of the brain. They will probably be new to most of our readers, and therefore we feel no hesitation in quoting Mr. Cooper's account.

"Dr. Foville, of Rouen, has also added some important facts to our knowledge, respecting the anatomy of the brain, and has supported his doctrines by numerous pathological facts.

In a demonstration which he gave at Guy's Hospital, in 1830, he stated, that he considered the corpus callosum was not produced by the transverse fibres of the hemispheres running across from one side to the other, forming a complete commissure, as stated by Gall and Spurzheim; but that it is produced by the corpora pyramidalia, after they have entered the corpora striata, rising up and forming a union in the middle line above the striated bodies and thalami—thus forming the corpus callosum; while another portion passing downwards and inwards, and then upwards to the middle line of the corpus callosum, where being only slightly separated from a similar structure or plane of medullary matter in the opposite side, produces the septum lucidum and fifth ventricle; while a third portion, passing laterally, goes to the hemispheres of the brain. To explain this better, it will be necessary to trace Dr. Foville's view from the spinal marrow; which is composed of two symmetrical portions, each formed of an anterior, posterior, and middle column.

The portion of the spinal marrow named the medulla oblongata, forms several enlargements; one part of which is prolonged into the cerebrum, another into the corpora quadrigemina, and a third into the cerebellum.

Thus the corpora restiformia, are traced to the cerebellum; the corpora olivaria, to the tubercula quadrigemina; and the corpora pyramidalia, to the cerebrum; the anterior fibres of the corpora pyramidalia decussating.

In the cerebellum, the corpus restiforme unites with the processus ad testes and the tuber annulare, forming a mass, which expands into a plane extending from within outwards, to the cineritious matter, where it lines all the folds formed in the cineritious exterior.

One part of this plane passes backwards, from without inwards; and with its fellow from the opposite side, forms a commissure in the processus vermiformis, analogous to the corpus callosum, or commissure of the cerebrum.

The corpora olivaria, form two bundles of fibres, which may be traced to the tubercula quadrigemina.

The corpora pyramidalia, each forms two bundles of fibres—the anterior of which only decussate; the posterior continue to the superior transverse fibres of the tuber annulare, and rest upon it, while their upper surface forms the floor of the fourth ventricle. These two bundles are separated from each other by the corpus niger, and proceed nearly parallel till they diverge in the corpora striata, and thalami nervorum opticorum; and form a large plane, directed obliquely outwards and upwards, of nearly a triangular figure, bounded by two straight and one curved line. The two sides of the expanded crus, or plane, form the two straight lines; the corpus striatum and thalamus, the curved line to the outer side of the ventricle—to which the expanded fibres diverge as to a circumference.

From this circumference three separate planes proceed, forming three layers, one above the other, at their origin.

The first or superior of these planes, rises on the outer side of the corpus striatum and thalamus, at first nearly vertically; it forms a slight convexity outwards, and then bending inwards horizontally towards the mesial line, unites with its fellow to form the corpus callosum.

The corpus callosum, therefore, proceeds from the crura cerebri, and has nothing to do with the hemispheres; they form a true commissure to the crura cerebri; but Dr. Foville observes, that he has not ascertained whether the fibres of the two sides cross or anastomose with each other.

The second plane or central one, immediately beneath the former, at first ascends parallel with, and applied to that of the corpus callosum; then it separates, and is reflected inwards, nearly in a vertical direction, until it reaches the cineritious substance of the most elevated part of the hemispheres, along its whole length, when the convex external and flat internal parts meet each other. It lines the grey matter, following all its folds in the form of a white layer, of which the fibrous structure is not so evident as that of the plane itself. The fibres of this layer, therefore, diverge towards the circumference, and converge towards the expansion of the crura, of which they are a continuation.

The third plane, is of less extent than the two preceding; it proceeds from the same line with them, and descends to the outer side of the inferior half of the grey substance of the corpus striatum, invests it below, and advancing inwards, meets the corresponding plane from the opposite side, and then ascends, forming the septum lucidum of the ventricles. Some of the fibres of this plane do not pass directly to the septum lucidum, but pass backward, reach the extremity of the cornu ammonis, and becoming continuous with the corpus fimbriatum, pass into the fornix, and form a second communication with the septum lucidum. Other fibres pass and form an expansion, especially destined for the temporal lobe of the hemispheres.

From these interesting anatomical points, Dr. Foville considers that the cerebellum is the central organ of sensation; the tubercula quadrigemina, of the influence of organic life; and the cerebrum, of motion; and that the mind resides more particularly in the cineritious matter, as a percipient centre: and considers the medullary part of the brain as the nerves, conveying the influence of the will from the sensorium to the exterior of the body, or parts of motion, and conveying to the sensorium the impressions of the exterior senses.

As physician to a large lunatic asylum at Rouen, Dr. Foville had an opportunity of examining frequently the brains of those who were the subjects of different kinds of madness; from which he was led to divide mental derangements into three classes. Those depending on false impressions, in which the medullary portions were found diseased, and hence their powers of conducting proper impressions deranged; those depending on a troubled state of mind itself, in which the cineritious portions were diseased; and in those depending upon paralysis of the insane, he found the cellular membrane connecting the fibres of the medullary part of the brain so altered by inflammation, that its structure could not be unravelled.

When extravasation of blood has occurred into the brain, Dr. Foville has seen paralysis recede as the blood became absorbed, and recur at intervals if the disease tended to farther extravasation, either of blood or serum. He also brought forward several cases, to prove that the motions of the lower extremities depended on the integrity of the thalami, and of the upper extremities on the integrity of the corpora striata; and one case in particular, where a surgeon, on putting on a ligature around the axillary artery, included some or one of the axillary nerves; the result of which was an abscess in the thalamus, and consequent death. In diseases extending from the corpora striata to the thalami, the lower extremities are first affected, and then the upper." 183.

In conclusion, we again express our warm approbation of Mr. Cooper's work, and can conscientiously recommend it to English students of anatomy.

